# Benchmarking clustering algorithms on estimating the number of cell types from single-cell RNA-sequencing data

**DOI:** 10.1186/s13059-022-02622-0

**Published:** 2022-02-08

**Authors:** Lijia Yu, Yue Cao, Jean Y. H. Yang, Pengyi Yang

**Affiliations:** 1 School of Mathematics and Statistics, University of Sydney, Sydney, NSW 2006 Australia; 2 Computational Systems Biology Group, Children’s Medical Research Institute, University of Sydney, Westmead, NSW 2145 Australia; 3 Charles Perkins Centre, University of Sydney, Sydney, NSW 2006 Australia

## Abstract

**Background:**

A key task in single-cell RNA-seq (scRNA-seq) data analysis is to accurately detect the number of cell types in the sample, which can be critical for downstream analyses such as cell type identification. Various scRNA-seq data clustering algorithms have been specifically designed to automatically estimate the number of cell types through optimising the number of clusters in a dataset. The lack of benchmark studies, however, complicates the choice of the methods.

**Results:**

We systematically benchmark a range of popular clustering algorithms on estimating the number of cell types in a variety of settings by sampling from the Tabula Muris data to create scRNA-seq datasets with a varying number of cell types, varying number of cells in each cell type, and different cell type proportions. The large number of datasets enables us to assess the performance of the algorithms, covering four broad categories of approaches, from various aspects using a panel of criteria. We further cross-compared the performance on datasets with high cell numbers using Tabula Muris and Tabula Sapiens data.

**Conclusions:**

We identify the strengths and weaknesses of each method on multiple criteria including the deviation of estimation from the true number of cell types, variability of estimation, clustering concordance of cells to their predefined cell types, and running time and peak memory usage. We then summarise these results into a multi-aspect recommendation to the users. The proposed stability-based approach for estimating the number of cell types is implemented in an R package and is freely available from (https://github.com/PYangLab/scCCESS).

**Supplementary Information:**

The online version contains supplementary material available at 10.1186/s13059-022-02622-0.

## Background

Single-cell RNA-sequencing (scRNA-seq) has emerged as a key technology for profiling the gene expression program at both the global transcriptome level and at the single-cell resolution. Effective computational analyses of scRNA-seq data is essential for extracting underlying biological knowledge from the large amount of data generated by such technology [1] and clustering techniques have been the workhouses especially in cell clustering and cell type discovery [2]. While much attention has been given to clustering cells into cell type groups, estimating the number of cell types in a given scRNA-seq dataset has received less attention. This is particularly noticeable in the literature, where only a small proportion [3] of the large number of clustering algorithms [4, 5] designed for cell clustering is capable of estimating the number of cell types. Estimating the number of cell types can be considered as finding the optimal number of clusters for a given scRNA-seq data with the assumption that each cluster corresponds to a unique cell type in the dataset [6]. Under this assumption, current clustering methods that estimate the number of cell types can be loosely classified into the following categories: (i) intra- and inter-cluster similarity, (ii) modularity in community detection, (iii) eigenvector-based metrics, and (iv) stability metrics. Given the lack of systematic evaluation of clustering algorithms on their performance on estimating the number of cell types, in this study, we set out to systematically assess the estimation of the number of cell types for a collection of clustering algorithms from each category summarised below.

Intra- and inter-cluster similarity is one of the most widely applied approaches for estimating the optimal number of clusters in a given dataset [7, 8]. This involves calculating indices that measure the closeness of items in each cluster and separations among clusters. In scRNA-seq data analysis, methods in this category include scLCA [9] which uses Silhouette index [10], CIDR [11] which uses Calinski-Harabasz (CH) index [12], and SHARP [13] that relies on both indices (Silhouette and CH) and hierarchical heights of the clustering to determine the number of clusters. RaceID [14] uses the Gap statistic [15], follows the idea of intra- and inter-cluster similarity but introduces a statistical test to compare within-cluster dispersion. Similarly, SINCERA [16] uses a minimum distance approach to obtain “non-singleton” cell clusters. The second category of community detection-based techniques mostly relies on the Louvain algorithm [17] and Leiden algorithm [18] to optimise community structure to find the best possible grouping. This strategy is implemented by a number of scRNA-seq clustering methods including ACTIONet [19], Monocle3 [20–22], and Seurat [23]. The third category involves using eigenvector-based techniques, where the methods typically apply eigengap heuristic to estimate the number of cell types [24]. Examples such as SIMLR [25] partition the data into a specific number of clusters that maximise the eigengap. In Spectrum [26], the authors extended the idea of eigengap heuristic and built a multimodality gap heuristic algorithm in which can be applied to Gaussian or non-Gaussian structures. Similarly, SC3 [27] partitions the data by examining the eigenvalue based on the Tracy-Widom test [28, 29]. Finally, clustering stability is another commonly employed metric for determining the number of clusters in the computation and data science literature [30, 31]. The intuition behind these approaches is that clustering output, generated under the optimal number of clusters, would lead to more stable or reproducible clusters compared to those generated under suboptimal number of clusters. An example in this fourth category is densityCut [32], which estimates the number of cell types from a given dataset by modelling the density of cell distributions for generating a hierarchical cluster tree and subsequently selecting clusters that are most stable in the hierarchical cluster tree. In this study, we propose an alternative stability-based approach by taking advantage of scCCESS, a random sampling-based ensemble deep clustering model, previously proposed for scRNA-seq data clustering [33] for estimating the number of cell types. Our key assumption is that clustering from using the optimal number of clusters would be the most robust to small perturbations in the data, such as those introduced by random resampling, compared to those generated under the suboptimal number of clusters.

**Fig. 1:**
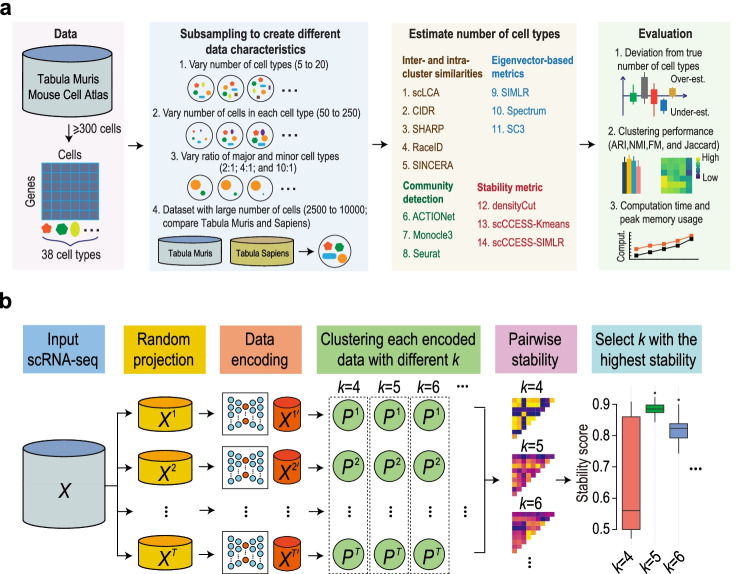
Schematic summaries of (**a**) benchmark workflow and (**b**) clustering stability measure. **a** Summary of the benchmark workflow. A panel of fourteen scRNA-seq clustering methods that perform the estimation of the number of cell types were evaluated under four main settings for creating different data characteristics via sampling from the Tabula Muris and Tabula Sapiens data. Evaluation includes deviation from the true number of cell types, clustering concordance with predefined cell type labels, and computational time and memory usage of each method. **b** Illustration of clustering stability, implemented as part of the single-cell Consensus Clusters of Encoded Subspaces (scCCESS) [33], for estimating the number of cell types in a given scRNA-seq dataset

Together with the current state-of-the-art methods and our proposed stability-based approach, we present a systematic and quantitative analysis of single-cell clustering algorithms, focusing on their performance on estimating the number of cell types. Specifically, we benchmark the above fourteen clustering methods from each of the four categories (Fig. 1a) across a large number of datasets sampled from the Tabula Muris project [34] representing different data characteristics in various settings. We evaluate the accuracy on determining the number of cell types, performance of cell clustering, and computing time and peak memory usage of each method on each of all datasets. We further cross-compared the performance of clustering algorithms on datasets with a large number of cells using both Tabula Muris and Tabula Sapiens data [35]. We summarise these findings into a multi-aspect recommendation to the users, and highlight potential areas requiring future research.

## Results

### Benchmark framework for clustering algorithms on estimating the number of cell types

To evaluate the performance of clustering methods on estimating the number of cell types from data with various characteristics (Fig. 1a), we subsampled from the Tabula Muris dataset [34] to create three main settings including (i) varying the number of true cell types from 5 to 20 (increment by 1) while fixing the number of cells in each cell type as 200; (ii) varying the number of cells from 50 to 250 (increment by 50) while fixing the number of cell types at 5, 10, 15, and 20; and (iii) varying the ratio of cells between major and minor cell types (i.e. 2:1, 4:1, and 10:1) while fixing the number of cell types at 10 and 20 (see the “Methods” for details). In particular, the number of cells is kept the same among all cell types in setting 1 and 2, whereas in setting 3, the number of cells is different between major and minor cell types. In addition, we also subsampled from both the Tabula Muris and the Tabula Sapiens [35] datasets to create a fourth setting in which datasets are with a large number of cells (2500 to 10,000). This last setting allows us to evaluate the performance of clustering methods on datasets with high cell numbers while also assessing if the findings are comparable across different species and data sources.

Among the fourteen methods compared in this benchmark study, we include twelve published clustering methods and two proposed stability-based methods. The two proposed methods use clustering stability extracted from scCCESS [33], an ensemble clustering algorithm, for the number of cell type estimation (Fig. 1b). Specifically, scCCESS samples multiple random projections from the original input scRNA-seq dataset and encodes the random projections to a lower dimension via autoencoders. Next, it clusters each encoded dataset and creates consensus from these clustering. We take advantage of the multiple clustering output generated from the encoded datasets for evaluating clustering stability of a cluster numbers *k* by employing scCCESS across a range of *k* values (by default *k* ∈ [2, *K*]; where *K* is the maximum number of clusters). Intuitively, the number of cell types in the dataset is determined by the *k* value that leads to the most stable clustering output among all encoded datasets. Thus, we calculate the pairwise agreement score of all clustering output on encoded datasets and select the *k* that gives the highest average score. Since scCCESS can be used with any clustering algorithm that allows user-specified *k* values, we coupled scCCESS with a basic *k*-means clustering algorithm and SIMLR [25], a single-cell specific clustering algorithm. We refer to them as scCCESS-Kmeans and scCCESS-SIMLR, respectively, thereafter.

### Overall performance of clustering algorithms on the number of cell type estimation

**Fig. 2:**
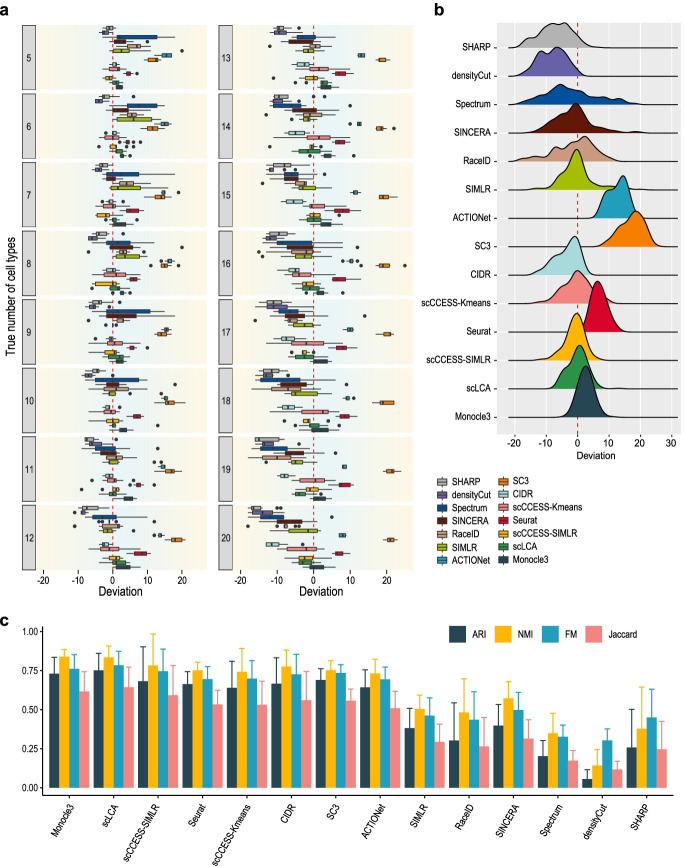
Overall performance and impact of cell type numbers on the number of cell type estimation. **a** Deviation of the estimated and the true number of cell types for each of the 14 clustering methods. Positive deviation represents over-estimation and negative deviation represents under-estimation. The true number of cell types ranges from 5 to 20 and each was repeated 10 times to capture the estimation variability. **b** Density plot summarising overall deviation across all numbers of cell types in **a**. **c** Concordance of clustering output and predefined cell type labels as quantified by four concordance measures. Each bar represents the average performance across datasets with 5 to 20 cell types, and error bars represent the standard deviation

We first compare each clustering method for correctly identifying the number of cell types by applying each method on 160 datasets that contain 5 to 20 cell types randomly sampled from the Tabula Muris dataset. The number of cells in each cell type was held constant at 200. Figure 2a shows for each method the deviations between the estimated number of cell types and the true number of cell types, with positive deviation representing over-estimation and negative deviation representing under-estimation. Across these datasets (i.e. sampled with 5 to 20 cell types), we found that Monocle3, scLCA, and scCCESS-SIMLR in general have a smaller median deviation compared to other methods, and, as expected, increasing the number of cell types in the dataset leads to higher under- and over-estimation. To summarise these results, we calculated the overall distribution of deviation across all datasets (Fig. 2b). The summary confirmed the performance of the above methods while highlighting the high instability found in methods such as Specturm, SINCERA, and RaceID, and the bias in underestimation (e.g. SHARP, densityCut) and overestimation (e.g. SC3, ACTIONet, Seurat).

Since a clustering method may correctly estimate the number of cell types in a dataset but still generate poor clustering of the cells, we next assessed the concordance between the clustering output and the predefined cell type labels (obtained from the original publication of Tabula Muris) using four evaluation metrics including Adjusted Rand Index (ARI), Normalized Mutual Information (NMI), Fowlkes-Mallows index (FM), and Jaccard index (Jaccard). The average clustering concordance and standard deviations across datasets sampled with different numbers of cell types were shown in Fig. 2c, and the detailed results are presented in Additional File 1: Fig S1. The assessment results from the four evaluation metrics are highly correlated. Overall, Monocle3, scLCA, scCCESS-SIMLR, CIDR, Seurat, and scCCESS-Kmeans show higher cell type clustering concordance with predefined cell type labels (≥ 0.5), in agreement with their better performance in estimating the number of cell types compared to other methods. Notably, however, higher cell clustering concordance does not necessarily mean a more accurate number of cell type estimation. For example, SC3 has comparatively high cell clustering concordance compared to other best performing methods (e.g. Monocle3) but was significantly over-estimating the number of cell types (Fig. 2a, b). These results highlight the importance of evaluating the number of cell types estimation accuracy of clustering algorithms independent of their performance on clustering cells.

### Impact of number of cells on number of cell type estimation and clustering

**Fig. 3:**
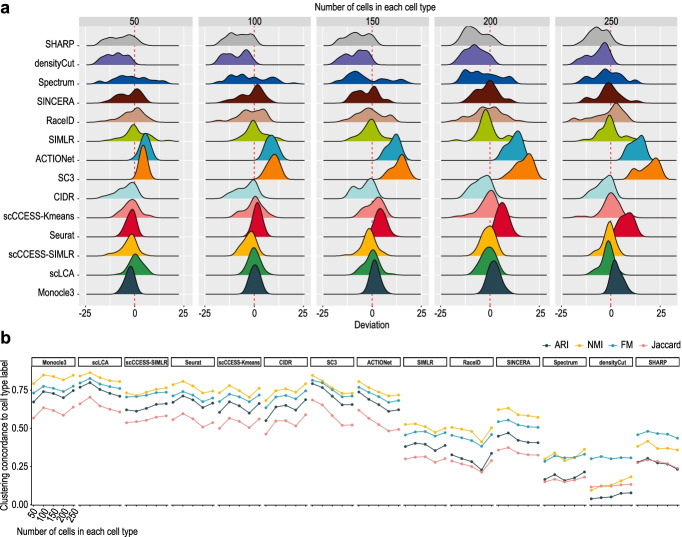
Impact of number of cells on the performance of number of cell type estimation. **a** Density plot summarising overall deviation in number of cell type estimation across all number of cell types (i.e. 5, 10, 15, and 20) with different number of cells in each cell type. **b** Concordance of clustering output and pre-defined cell type labels quantified by four concordance measures for datasets with different number of cells in each cell type (i.e. 50, 100, 150, 200, and 250). Each dot represents the mean value of concordance score

Besides comparing the performance of clustering algorithms on datasets with a fixed number of cells (i.e. 200) in each cell type, we examine the impact of the number of cells on the number of cell type estimation and the clustering of cells. To this end, we varied the number of cells in each cell type from 50 to 250 (increments by 50 in each test) and assessed the accuracy of the estimated number of cell types when the true number of cell types were set as 5, 10, 15, and 20. We found that, in general, increasing the number of cells helps most clustering algorithms reduce the variability in the number of cell type estimation but have a limited impact on their estimation deviation (Fig. 3a). However, SC3, ACTIONet, and Seurat are a few exceptions, showing a clear increase in variability in their number of cell type estimation on datasets with larger numbers of cells. A closer look at these results suggests that, interestingly, while most clustering algorithms show a similar level of deviation in the number of cell type estimation, SC3, ACTIONet, and Seurat, to a lesser degree, tend to over-estimate when the number of cells in each cell type increases (Fig. 3a and Additional File 1: Fig S2).

On the clustering of cells, we found that the performance in terms of clustering concordance to the pre-defined cell labels does not necessarily increase with an increasing number of cells in each cell type (Fig. 3b; Additional File 1: Fig S3-S4). In fact, the performance on cell clustering deteriorates for many clustering algorithms when the number of cells increases. This is particularly prominent for SC3 and ACTIONet, probably due to these methods considerably over-estimate the number of cell types when the number of cells increases in the datasets (Fig. 3a).

Together, these results suggest that methods such as SC3, ACTIONet, and Seurat tend to over-estimate the number of cell types, especially when the number of cells is large in each cell type, and also having higher variability in their estimation, unveiling a limitation of these clustering methods when dealing with datasets with a relatively large number of cells.

### Bias analysis of clustering algorithms on estimating the number of cell types

**Fig. 4:**
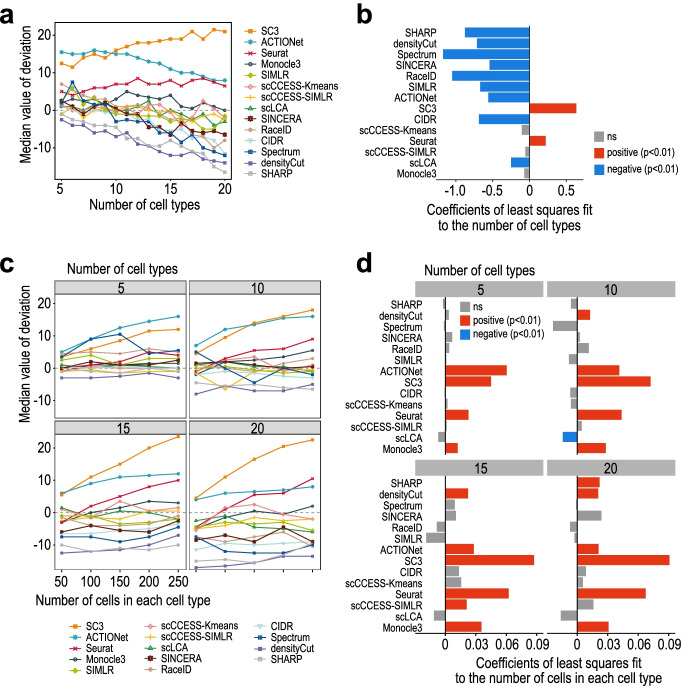
Bias analysis for each clustering algorithm on number of cell type estimation. **a** Median deviation values of each clustering algorithm on datasets with different number of cell types. **b** Coefficients of least squares fit to the median deviation value with respect to the number of cell types. **c** Median deviation values of each clustering algorithm on datasets with different number of cells (ranging from 50 to 250) in each cell type. Results are split by the number of cell types (i.e. 5, 10, 15, and 20). **d** Coefficients of least squares fit to the median deviation value with respect to the the number of cells in each cell type

Given the tendency of consistent under- and over-estimation of the number of cell types we noticed in some clustering algorithms in the benchmarking results, we set out to analyse if there is a systematic bias in the number of cell type estimation for each method and how that is confounded by the number of cell types and the number of cells per cell type in the datasets. Notably, we found while most clustering algorithms tend to under-estimate the number of cell types when the true number of cell types increases in the datasets (e.g. densityCut, SHARP, Spectrum, CIDR), SC3 and Seurat, counter-intuitively, appear to over-estimate when applied to data with larger cell type numbers (Fig. 4a).

The analysis of the coefficients (slopes) of the linear regression lines fitted to the number of cell types estimated by each method confirms these observations (Fig. 4b). Monocle3, scCCESS-Kmeans, and scCCESS-SIMLR are the only methods that do not show bias with respect to the changing number of cell types (Fig. 4b). In addition, while some methods display similar deviation irrespective of the changes in the number of cells in each cell type (e.g. RaceID, SINCERA, SIMLR), we found that, again, several methods, especially SC3, ACTIONet, and Seurat, increasingly over-estimate cell type number when the number of cells per cell type increases (Fig. 4c, d). These results highlight the existence of systematic biases in some of the clustering algorithms in the number of cell type estimation, and the biases are compounded by both the number of cell types and the number of cells per cell type in the datasets. In particular, the number of cell types in a dataset could have an opposite effect on their estimation depending on which clustering algorithm was used, and methods such as SC3 and Seurat tend to significantly over-estimate the cell type numbers when applied to datasets under two settings (i) with a large number of cell types, and (ii) a large number of cells in each cell type.

### Impact of imbalance of cell ratios on number of cell type estimation and clustering

While the previous experiments simulated datasets with different numbers of cell types and also different numbers of cells in each cell type, the number of cells among all cell types was kept the same. As such, these settings facilitate the isolation and testing of the performance of clustering algorithms conditioned on these two key aspects (e.g. number of cell types; number of cells) on estimating the number of cell types, most scRNA-seq experiments generate data that capture cell types with different number of cells, sometimes with highly imbalanced ratios. To test the impact of imbalanced ratios of cells among different cell types, we set out subsampling from Tabula Muris data to create major and minor cell types (each with 5 or 10 cell types) with imbalanced cell ratios of 2:1, 4:1, and 10:1 (see the “Methods” section for details).

**Fig. 5:**
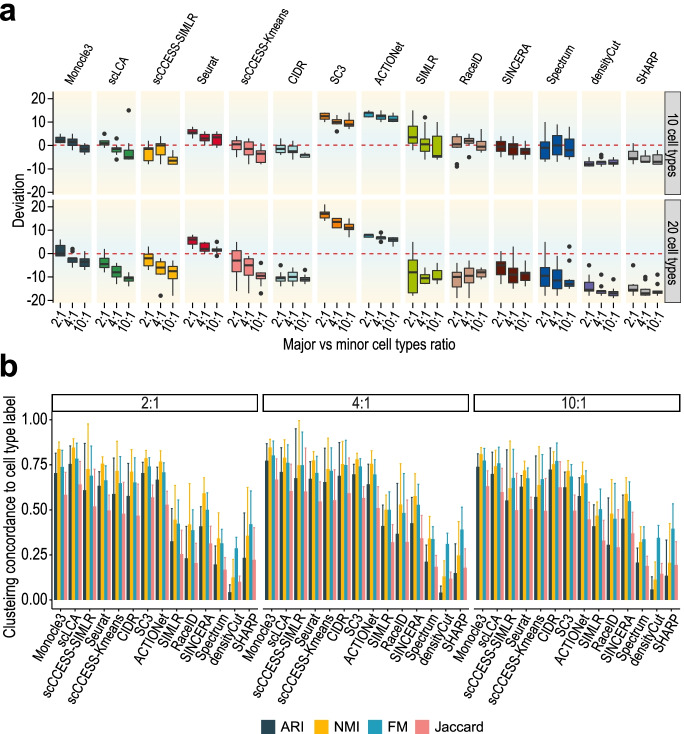
Impact of imbalance ratios of cells among cell types on the performance of the number of cell type estimation. **a** Deviation of the estimated and the true number of cell types for each clustering method on datasets with different imbalance ratios (i.e. 2:1, 4:1, and 10:1) and different number of cell types (i.e. 10 and 20). Each combination was repeated 10 times for estimating variability. **b** Concordance of clustering output and predefined cell type labels as quantified by four concordance measures for datasets with different imbalance ratios of 2:1, 4:1, and 10:1, and error bars represent the standard deviation

Figure 5a shows the performance on the number of cell type estimation in each imbalanced setting for each of the 14 clustering algorithms, and Fig. 5b quantifies the concordance of cell clustering to cell type labels averaged across the results from 10 and 20 cell types. We found that the imbalanced ratio of cells in major and minor cell types in general leads to a reduction in the estimated number of cell types when the ratio increases (Fig. 5a). This unintended reduction helps methods that tend to over-estimate the number of cell types (e.g. Seurat, SC3, ACTIONet) but results in under-estimation for others (e.g. scLCA, scCCESS-Kmeans, scCCESS-SIMLR). Again, the performance on cell clustering does not always match the accuracy of the number of cell type estimation (e.g. Seurat) (Fig. 5b and Additional File 1: Fig S5). These results suggest that the imbalance ratio of cells in major and minor cell types has an uneven impact on different clustering algorithms and the number of cell types (e.g. 10 and 20) in a dataset tends to be the key driver on their performance of the number of cell type estimation.

### Cross-comparison on datasets with high cell numbers using Tabula Muris and Tabula Sapiens data

**Fig. 6:**
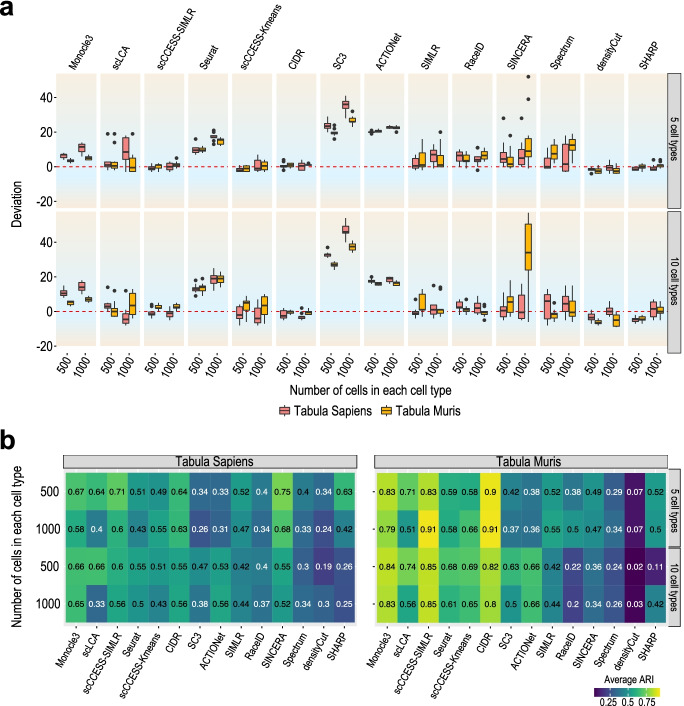
Performance of clustering methods on datasets with large numbers of cells sampled from Tabula Sapiens and Tabula Muris. **a** Deviation of the estimated and the true number of cell types for each clustering method on datasets with different number of cells (i.e. 2500 and 10000) depending on the number of cell types (i.e. 5 and 10) and the number of cells in each cell type. Each combination was repeated 10 times for estimating variability. **b** Concordance of clustering output and predefined cell type labels as quantified by ARI for each method

We further cross-compared the performance of different clustering algorithms on datasets with a large number of cells using both Tabula Muris and Tabula Sapiens data. Again, we observed clear overestimations of the number of cell types from SC3, ACTIONet, and Seurat (Fig. 6a) on these datasets, confirming the results from the bias analysis using datasets with increasing numbers of cells (Fig. 4c, d). When the overestimations are extreme, the performance of cell clustering from these methods do suffer (Fig. 6b and Additional File 1: Fig S6). Nevertheless, methods that do not show large over or underestimation of number of cell types do not necessary perform better than those that significantly overestimate. The most striking examples include densityCut, Spectrum, and RaceID. Finally, with few exceptions (e.g. SINCERA), the performance of different clustering algorithms on Tabula Muris and Tabula Sapiens datasets are highly consistent across the number of cell types and number of cells tested in our experiments (Fig. 6 and Additional File 1: Fig S6). These results confirm that the performance assessments are generalisable across datasets from different species and sources.

### Running time and peak memory usage

**Fig. 7:**
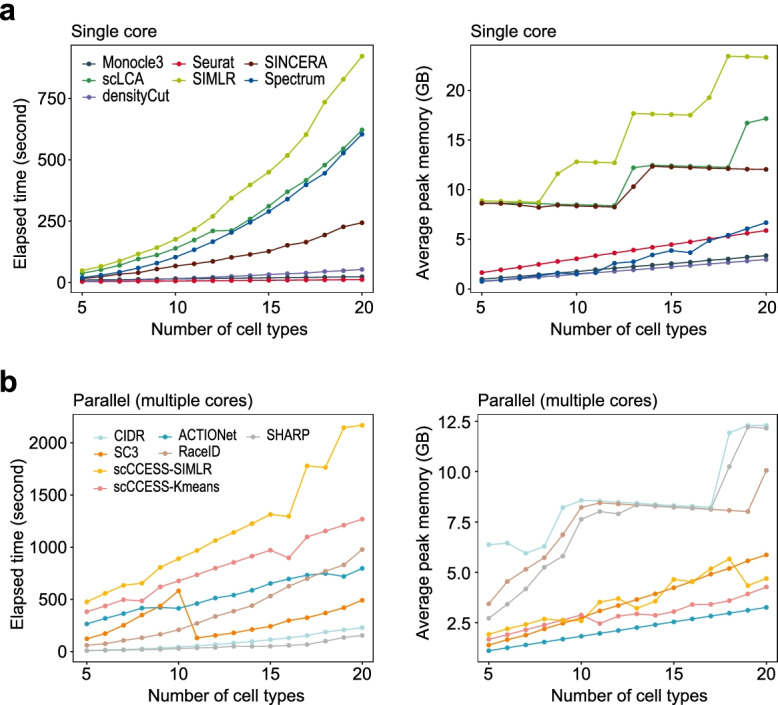
Benchmark of the elapsed time and peak memory usage across datasets with different number of cell types, ranging from 5 to 20. **a** The running time and peak memory usage of methods that uses only a single CPU core (i.e. densityCut, scLCA, SIMLR, Monocle3, Seurat, Spectrum, and SINCERA). **b** The running time and peak memory usage of methods that employ multiple cores for parallel computing (i.e. ACTIONet, RaceID, scCCESS-Kmeans, SHARP, CIDR, SC3 and scCCESS-SIMLR)

Lastly, we benchmarked the computational time and peak memory usage for all clustering methods in each of the four settings (i) varying the number of cell types (Fig. 7), (ii) varying the number of cells in each cell type (Additional File 1: Fig S7); (iii) varying the imbalanced ratio of cells among cell types (Additional File 1: Fig S8), and (iv) datasets with large number of cells (Additional File 1: Fig S9). Seven out of 14 methods (densityCut, scLCA, SIMLR, Monocle3, Seurat, Spectrum, and SINCERA) use only a single thread to perform clustering analysis, whereas the remainders run on parallel computing mode that utilise multiple CPU cores, if available, by default. In general, clustering on large datasets needs more computing time and uses more memory in all settings (e.g. increasing number of cell types, increasing number of cells), and, interestingly, several single thread methods take shorter computing times than most parallel methods, and on average, more peak memory usage than parallel methods (Fig. 7).

Specifically, on computational time, three single thread methods (Seurat, Monocle3, densityCut) and two parallel methods (CIDR, SHARP) greatly outperform others, where the clustering on any benchmark datasets were completed in less than 200 s. scLCA is one of the top-performing methods on the number of cell type estimation but uses significantly more time compared to other faster methods, revealing a trade-off between estimation accuracy and computational efficiency for this method. As expected, ensemble-based clustering methods (i.e. scCCESS-Kmeans and scCCESS-SIMLR) take a longer time to complete, since multiple clustering needs to be performed and then combined to get a consensus output. Nevertheless, when dealing with datasets with a large number of cells, RaceID appears to be significantly slower than all other methods (Additional File 1: Fig S9).

On memory usage, SIMLR is the most memory-consuming method compared to others and may prohibit its application to large-scale datasets (Fig. 7 and Additional File 1: Fig S9). However, scCCESS-SIMLR reduces the feature dimension of the input data and therefore significantly reduces the peak memory usage. CIDR is reputable for its ultra-fast clustering time, but uses the highest amount of peak memory among all parallel methods, highlighting a trade-off between memory usage and computational efficiency for this method.

## Discussion

**Fig. 8:**
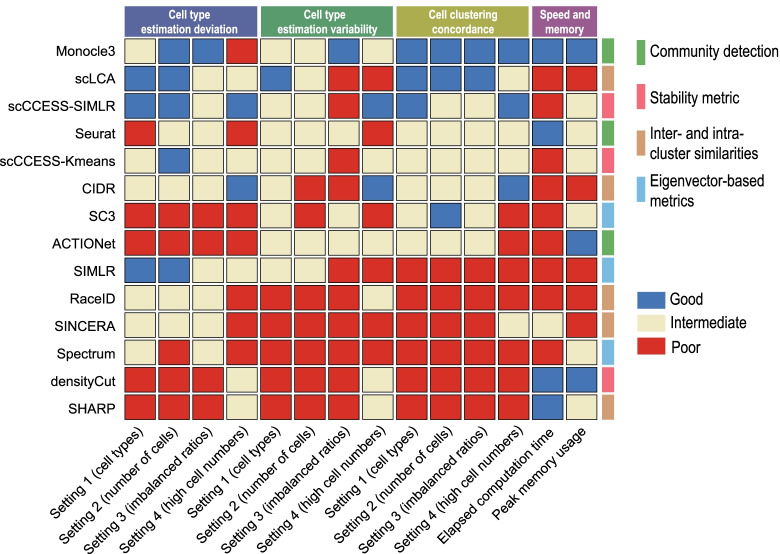
Summary of clustering method performance across all major evaluation criteria. Criteria and cutoff values for performance categories are described in detail in the Method section. Clustering methods are ranked by their overall performance across all criteria

Overall, we observed that methods based on community detection and clustering stability performed more favourably than methods from other categories across most of the evaluation criteria (Fig. 8). Eigenvector-based methods, in comparison, performed unfavourably in general and methods based on inter- and intra-cluster similarities show a very broad range of performance from those that performed very well (i.e. scLCA) to those poorly (i.e. SHARP and RaceID). These findings are largely consistent with a recent study reporting that a stability-based clustering method performed the best while methods such as SC3 overestimates the number of cell types in many cases and RaceID takes more computation time than alternatives such as Seurat [36]. While it is hard to pinpoint the factors contributing to the performance difference among different categories of clustering algorithms, we suspect that methods based on community detection and clustering stability may share a similar implementation strategy whereas the implementation strategies for methods based on inter- and intra-cluster similarities are more diverse and hence may have contributed to the wider range of performance. Having said that, these results do indicate that, while there may be a general trend in performance for certain categories of methods, the specific implementation of each method also plays a significant role in determining its performance in each of these evaluated aspects. Furthermore, these results also demonstrate that, while there is an overall concordance in performance across most of the evaluation criteria, each criterion does shed a unique light on a specific aspect of each clustering algorithm. Most importantly, we found that better performance on cell clustering does not necessarily imply accuracy in estimating the number of cell types. This could happen when a method is able to correctly estimate the number of cell types even when its clustering of cells to their respective groups is less precise. These results highlight the importance of evaluating the number of cell types independently when cell type detection is the main goal in scRNA-seq data analysis.

Although the sampling framework we used in this study creates scRNA-seq data with discrete cell types from the Tabula Muris resource, cell type structures from many biological systems are hierarchical with subpopulations of cells residing in each major cell type [37]. Related to this, the improving capability of scRNA-seq for profiling complex tissues and organs has led to datasets with increasing numbers of cell types and potentially further compounded by hierarchical cell type relationships. Thus, developing frameworks that are capable of benchmarking multi-resolution or multi-scale clustering on datasets with large numbers of cell types is a critical future research direction. Another recent expansion in single-cell omics field is the increasing availability of multimodal single-cell omics data produced by new enabling sequencing technologies such as single-cell Assay for Transposase Accessible Chromatin using sequencing (scATAC-seq) [38] and cellular indexing of transcriptomes and epitopes by sequencing (CITE-seq) [39] among others. Therefore, integrative methods, such as multi-view clustering [40, 41], that use several omics types generated from the same set of samples have the potential to reveal information, including hierarchical cell type structure, that are not accessible by examining only a single data type. Evaluating clustering algorithms for their performance on estimating the number of cell types from multimodal datasets is challenging and requires further methodological innovation.

## Conclusions

Clustering is an essential technique for scRNA-seq data analysis. While a tremendous amount of work has been done for designing and evaluating algorithms for clustering cells into cell types, their performance on estimating the number of cell types from a scRNA-seq dataset is yet to be systematically assessed. In this study, we have benchmarked 14 scRNA-seq clustering methods on estimating the number of cell types in datasets with various characteristics. We have also assessed other related aspects of performance including cell clustering and their scalability in terms of running time and memory usage. We compiled these multi-faceted evaluations into a recommendation (Fig. 8), summarising the performance of each of the methods according to each evaluation criteria. We expect this work will foster future development of scRNA-seq data clustering methods by providing a reference to the performance on the estimation of number of cell types in such data.

## Methods

### Sampling of Tabula Muris and Tabula Sapiens data

To create datasets with varying but well-defined characteristics, we took advantage of the large number of cells and cell types profiled by the Tabula Muris project [34] by subsampling from this dataset different numbers of cells and types and creating different imbalance ratios (described below). Specifically, Tabula Muris dataset contains 53,760 cells (FACS sorted and sequenced using Smart-Seq2 protocol) from 81 cell types of 20 organs of 7 mice. Cell types that include no more than 300 cells were excluded from this study. Overall, 38 cell types with 39,712 cells and 23,433 genes were included in the selection pool after filtering.

Four settings were considered in this benchmark study. The first is to benchmark the performance of clustering algorithms on estimating the number of cell types in datasets that contain different numbers of cell types. In particular, we held the number of cells in each cell type at a constant of 200, while varying the number of cell types by randomly sampling from all 38 candidate cell types in Tabula Muris dataset 200 cells each from 5 to 20 cell types (by step of 1). We repeated this sampling procedure 10 times where 10 datasets were sampled for each number of combination for the purpose of estimating variability, resulting in a total of 160 datasets (i.e. (20 − 4)×10).

In the second setting, we benchmarked the impact of different numbers of cells in each cell type on the number of cell type estimation. In this setting, we set the number of cell types in a dataset to be 5, 10, 15, and 20 by sampling from the 38 cell types in the Tabula Muris dataset and varied the number of cells in each cell type from 50 to 250 (increment by 50). Similar to above, we repeated the sampling 10 times for each number of cell type and number of cell combination, resulting in a total of 200 datasets (i.e. 4 × 5 × 10).

In the third setting, we tested the impact of datasets with different imbalance ratios by creating major and minor cell types that contain different numbers of cells. First, we sampled from Tabula Muris dataset 10 or 20 cell types and divided them into two equal groups for creating major and minor cell types (i.e. 5 or 10 cell types each for 10 and 20, respectively). We then created major and minor cell types by setting the number of cells in the major cell types as 200 and varying the number of cells in the minor cell types as 100, 50, and 20, leading to imbalance ratios of 2:1, 4:1, and 10:1 for the major and minor cell types. Again, we repeated the sampling 10 times, resulting in 60 datasets (i.e. 2 × 3 × 10).

To assess the performance of clustering algorithms on datasets with large number of cells while also validating if the benchmark results obtained from using Tabula Muris datasets are consistent compared to datasets sampled from another source, in the last setting, we sampled from Tabula Sapiens data [35] to create datasets with either five or ten cell types and each with either 500 or 1000 cells in each cell type. These lead to datasets with 2500 to 10,000 cells. The same sampling procedures were repeated on the Tabula Muris data to create datasets with the matching sizes so the performance of each clustering algorithm on the datasets with same sizes can be compared across different data sources. As above, we repeated the sampling 10 times, resulting in 80 datasets with large numbers of cells (i.e. 2 × 2 × 2 × 10).

Finally, all subsampled datasets are un-normalised raw count matrices. For methods that require normalised and/or log-transformed count matrix, we converted the raw count matrix to log-normalised count matrix using the ‘scater’ [42] package.

### Proposed clustering stability-based approach for number of cell type estimation

Building on single-cell Consensus Clusters of Encoded Subspaces (scCCESS), an ensemble clustering algorithm we developed previously [33], here we propose a clustering stability-based approach for the number of cell type estimation. scCCESS implements an autoencoder-based cluster ensemble for single cell clustering. It first generates multiple random projections from the original input scRNA-seq dataset and trains a collection of autoencoders, unsupervised deep learning neural networks, each on a random projection. It then encodes the data to multiple low dimensional data from which multiple clustering outputs are generated for creating the ensemble. We hypothesised that the number of cell types in a dataset is best estimated when multiple clustering output, each from a random project and dimension reduction, are highly concordant with each other (i.e. high stability). To this end, we take advantage of the multiple clustering output generated from the collection of autoencoders by quantifying stability of these clustering results across a range of *k* values using either *k*-means or SIMLR [25] clustering algorithms and use the *k* with the highest overall stability score (median of all pairwise concordance scores measured using normalised mutual information [NMI]) as the estimation of the number of cell types in an input scRNA-seq dataset.

### Clustering methods that estimate the number of cell types

We examined eleven established single-cell clustering approaches as well as two clustering stability-based methods that we proposed above. The methods were chosen from the scRNA-tools database [43], representing a wide range of popular clustering algorithms used for cell clustering and the number of cell type estimation from scRNA-seq data (Fig. 1a). Table 1 summarises the details of each approach, including the version of the code utilised in this benchmark analysis and its publication.

**Table 1:** scRNA-seq clustering methods for number of cell type estimation evaluated in this study

Methods	Platform	Clustering type	Category	Ref.	Version
Monocle3	R	Leiden clustering	Community detection	[22]	0.2.3.0
scLCA	R	Spectral clustering	Intra- and inter-cluster similarity	[9]	0.0.0.9
scCCESS-SIMLR	R	Ensemble of SIMLR	Stability metric	[33]	0.0.1
ACTIONet	R/C++	Leiden clustering	Community detection	[19]	2.0.18
Seurat	R	Louvain clustering	Community detection	[23]	4.0.1
scCCESS-Kmeans	R	Ensemble of K-means	Stability metric	[33]	0.0.1
CIDR	R	Hierarchical clustering	Intra- and inter-cluster similarity	[11]	0.1.5
SC3	R	Hierarchical clustering	Eigenvector-based metrics	[27]	1.18.0
SIMLR	R	Spectral clustering	Eigenvector-based metrics	[25]	1.18.0
RaceID	R/C++	K-means	Intra- and inter-cluster similarity	[14]	0.2.3
SINCERA	R	Hierarchical clustering	Intra- and inter-cluster similarity	[16]	0.99.0
Spectrum	R	Spectral clustering	Eigenvector-based metrics	[26]	1.1
densityCut	R	Hierarchical clustering	Stability metric	[32]	0.0.1
SHARP	R	Meta-clustering	Intra- and inter-cluster similarity	[13]	1.1.0

All parallel computing methods, including RaceID, CIDR, ACTIONet, scCCESS, SC3, and SHARP, were benchmarked by using eight cores. For methods such as scCCESS, SIMLR, scLCA, and Spectrum that allow explicit specification of the range of *k* values to be evaluated, we tested these from 2 to the upper bound of 25 for estimating the number of cell types. For scCCESS, the ensemble size was set as 20. When using SIMLR, we set the principal component as 15 and used ‘SIMLR_Large_Scale()’ function to avoid the data size limitation problem on ‘SIMLR()’ function. All other parameters of each method were set as the default values. To benchmark the elapsed time and peak memory usage, we evaluate all processing steps of each method, including gene and cell filtering if they are part of clustering steps implemented in a clustering package.

### Cell clustering evaluation metrics

To benchmark the cell clustering results from the four settings, four evaluation measures were employed to quantify the concordance of clustering results on each scRNA-seq dataset with respect to their predefined cell-type annotations [44]. These included adjusted Rand index (ARI), normalised mutual information (NMI), Fowlkes–Mallows index (FM), and Jaccard index (Jaccard).

Let *S* be a set of N cells, then a clustering **U** on *S* is a way of partitioning *S* into non-overlap subset{*U*_1_, *U*_2_, ⋯, *U*_*R*_}, where ${\bigcup}_{i=1}^R{U}_i=S$ and *U*_*i*_ ∩ *U*_*j*_ = ∅ for *i* ≠ *j*. Here, we define **U** = {*U*_1_, *U*_2_, ⋯, *U*_*R*_} as the gold standard cell type labels, **V** = {*V*_1_, *V*_2_, ⋯, *V*_*c*_} is a partition generated by a clustering. Pair counting based measures can be used for counting pairs of items on which the partition **U** and **V** agree or disagree. Specifically, the $\left(\genfrac{}{}{0pt}{}{N}{2}\right)$ item pairs in *S* can be classified into one of the four types: (i) *N*_11_: the number of pairs that are in the same partition in both **U** and **V**; (ii) *N*_00_: the number of pairs that are in different partitions in both **U** and **V**; (iii) *N*_01_: the number of pairs that are in the same partition in **U** but in different partitions in **V**; (iv) *N*_10_: the number of pairs that are in different partitions in **U** but in the same partition in **V**. Following this, ARI, NMI, FM, and Jaccard can be defined as follows [45, 46]:

$$\mathrm{ARI}\left(\mathbf{U},\mathbf{V}\right)=\frac{2\left({N}_{00}{N}_{11}-{N}_{01}{N}_{10}\right)}{\left({N}_{00}+{N}_{01}\right)\left({N}_{01}+{N}_{11}\right)+\left({N}_{00}+{N}_{10}\right)\left({N}_{10}+{N}_{11}\right)}$$

$$\mathrm{NMI}\left(\mathbf{U},\mathbf{V}\right)=\frac{I\left(\mathbf{U};\mathbf{V}\right)}{H\left(\mathbf{U}\right)+H\left(\mathbf{V}\right)}$$

$$\mathrm{FM}\left(\mathbf{U},\mathbf{V}\right)=\sqrt{\left(\frac{N_{11}}{N_{11}+{N}_{01}}\right)\left(\frac{N_{11}}{N_{11}+{N}_{10}}\right)}$$

$$\mathrm{Jaccard}\left(\mathbf{U},\mathbf{V}\right)=\frac{N_{11}}{N_{11}+{N}_{10}+{N}_{01}}$$

where *I*(***U***; ***V***) is the mutual information between **U** and **V**, defined as

$$I\left(\boldsymbol{U};\boldsymbol{V}\right)=\sum_{i=1}^R\sum_{j=1}^C\frac{\left|{U}_i\cap {U}_j\right|}{N}{\log}_2\frac{N\left|{U}_i\cap {V}_j\right|}{\left|{U}_i\right|\left|{V}_j\right|}$$

and H(∙) is the entropy of partitions, in which *H*(**U**) and *H*(**V**) are calculated

$$H\left(\mathbf{U}\right)=-\sum_{i=1}^R\frac{\left|{U}_i\right|}{N}\log\frac{\left|{U}_i\right|}{N}$$

$$H\left(\mathbf{V}\right)=-\sum_{j=1}^C\frac{\left|{V}_j\right|}{N}\log\frac{\left|{V}_j\right|}{N}$$

### Assessment of the run time and peak memory usage

All benchmark tasks were allocated on a research server with dual Intel(R) Xeon(R) CPU E5-2637 v4 @ 3.50GHz processor (16 cores and 64 Gb total memory). The elapsed run time was evaluated by the R function ‘system.time()’; timings for each method include all pre-processing steps. The usage of peak memory was monitored by R function ‘gc()’. The same seed was set for all steps involving stochasticity (i.e. dimension reduction and clustering) in each evaluating task.

### Performance summary criteria

Figure 8 summarises the performance of the evaluated methods across four criteria categories, including (i) deviation of the estimated number of cell types from the ground truth, which assesses the ability to estimate the number of cell types under the four settings; (ii) variability of the number of cell type estimation, which evaluate the variability of the estimated number of cell types across the four settings; (iii) cell clustering concordance with respect to the predefined cell type labels in the four settings, and (iv) the average speed and memory required for clustering across the four settings. For each metric, the performance of each method is considered as “good”, “intermediate”, or “poor”. Here, we list the criteria used to categorise the methods for each evaluation metric.

1. Deviation of the estimated number of cell types compared to the ground truth, defined as $\frac{\left(\# predicted\_ cell\_ types-\# true\_ cell\_ types\right)}{\# true\_ cell\_ types}$:

- Good: the deviation from the true number of cell types is ≤ 20%
- Intermediate: the deviation from the true number of cell types is 20% ≤ 50%
- Poor: the deviation from the true number of cell types is ≥ 50%

2. Variability of the number of cell type estimation:

- Good: the standard deviation is ≤ 2 cell types
- Intermediate: the standard deviation is 2 ≤ 5 cell types
- Poor: the standard deviation is ≥ 5 cell types

3. Clustering concordance based on 4 concordance metrics (range from 0 to 1), evaluating the clustering concordance from the predefined cell type labels:

- Good: the average value of metrics score is ≥ 0.7
- Intermediate: the average value of metrics score is 0.5≤ 0.7
- Poor: the average value of metrics score is ≤ 0.5

4. Speed, summarising of running time of each method:

- Good: the average running time for clustering a single dataset is ≤ 120 s
- Intermediate: the average running time for clustering a single dataset is 120 s ≤ 360 s
- Poor: the average running time for clustering a single dataset is ≥ 360 s

5. Memory and summary of peak memory usage of each method:

- Good: the average peak memory usage for clustering a single dataset is ≤ 4Gb
- Intermediate: the average peak memory usage for clustering a single dataset is 4Gb ≤ 8Gb
- Poor: the average peak memory usage for clustering a single dataset is ≥ 8Gb

## Supplementary Information

**Additional file 1.** Supplementary figures. Contains Fig S1-S9.

**Additional file 2. **Review history.

## Data Availability

Data used in this study were obtained from the Tabula Muris project [34] and the Tabula Sapiens project [35]. Source code of scCCESS, the proposed stability-based approach for estimating the number of cell types from scRNA-seq data, is deposited in Zenodo (DOI: 10.5281/zenodo.5899710) and is freely available from (https://github.com/PYangLab/scCCESS) under the open-source GPL-3 license [47].
