# Supplementary material for: Benchmarking clustering algorithms on estimating the number of cell types from single-cell RNA-sequencing data
Source: Genome Biol. 2022 Feb 8;23:49. doi: 10.1186/s13059-022-02622-0 (PMC8822786; doi:10.1186/s13059-022-02622-0)
Supplement: Supplementary file 1 — Additional file 1. Supplementary figures. Contains Fig S1-S9. [file 13059_2022_2622_MOESM1_ESM.pdf]

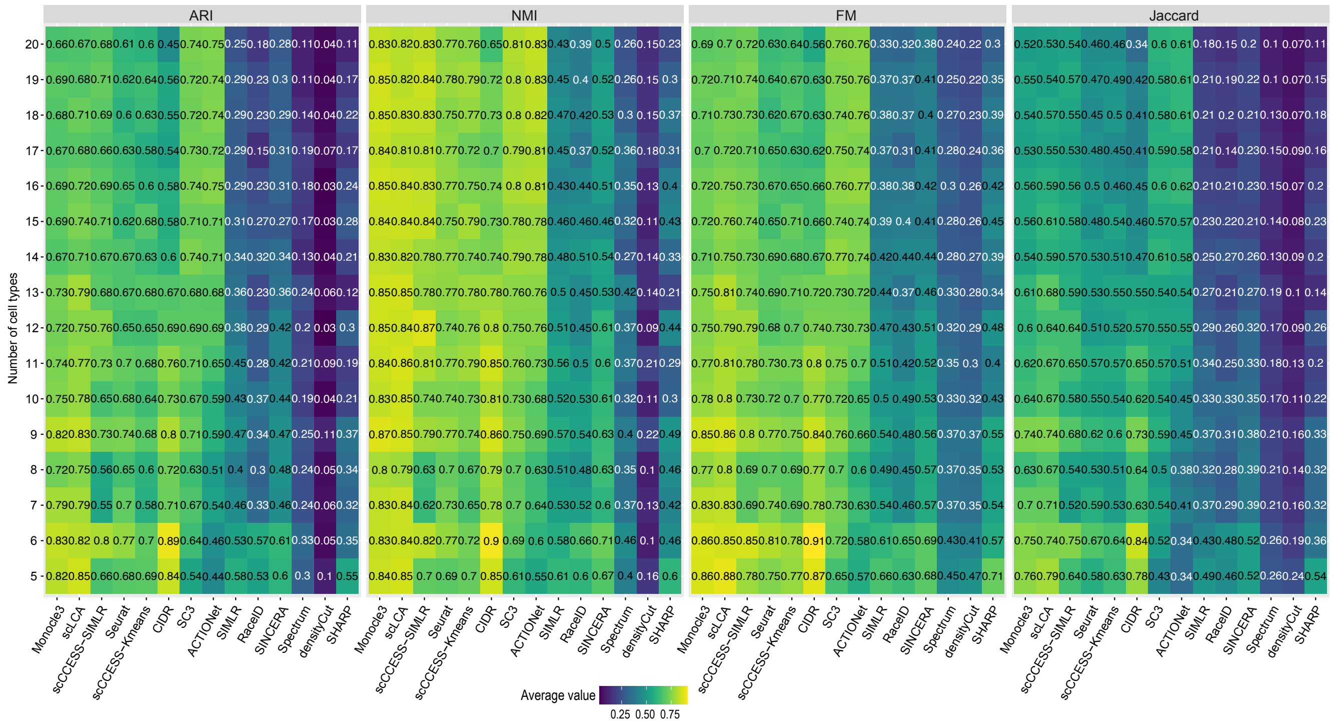

Fig S1. Concordance of clustering output and pre-defined cell type labels as quantified by four concordance measures, true number of cell types ranges from 5 to 20. Each tile represents the average score across 10 tests.

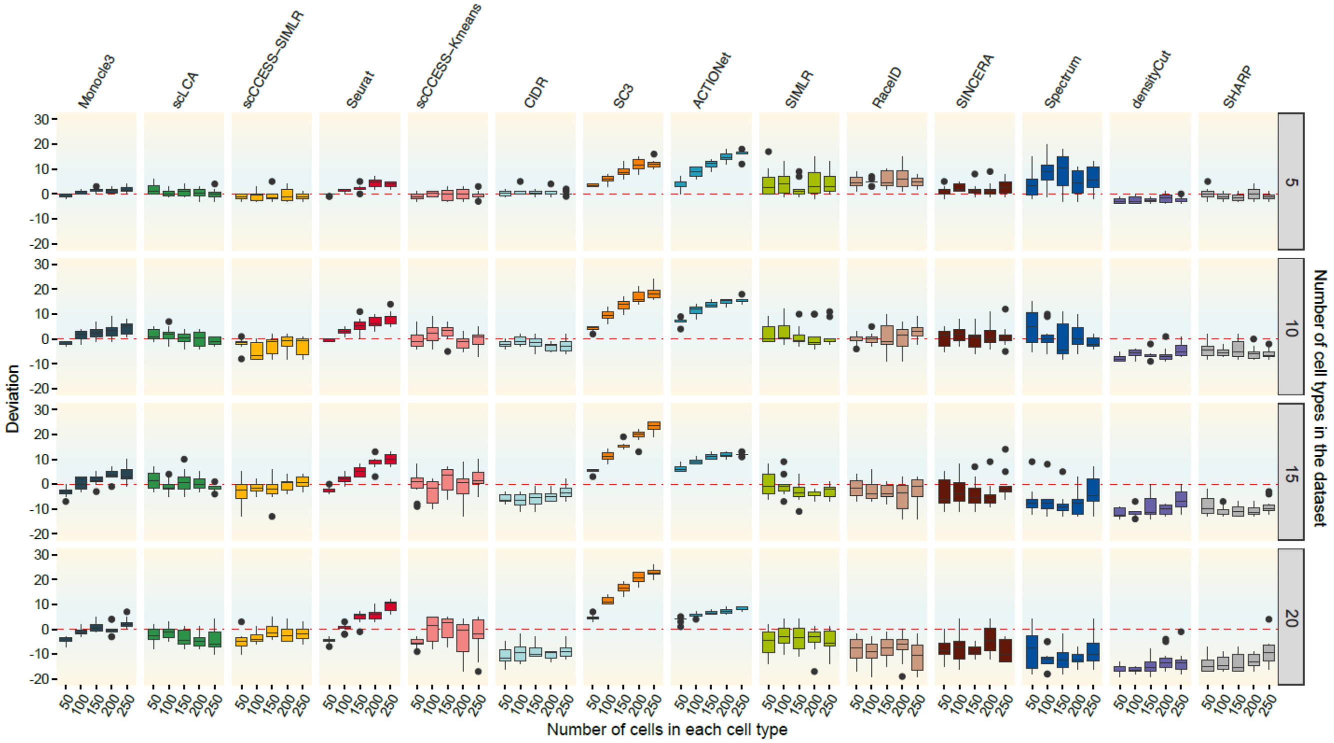

Fig S2. Deviation of the estimated and the true number of cell types for each of the 14 clustering methods on datasets with different number of cell types (i.e. 5, 10 ,15, and 20) and different number of cells in each cell type (i.e. 50, 100, 150, 200, and 250). Each combination was repeated 10 times for estimating variability.

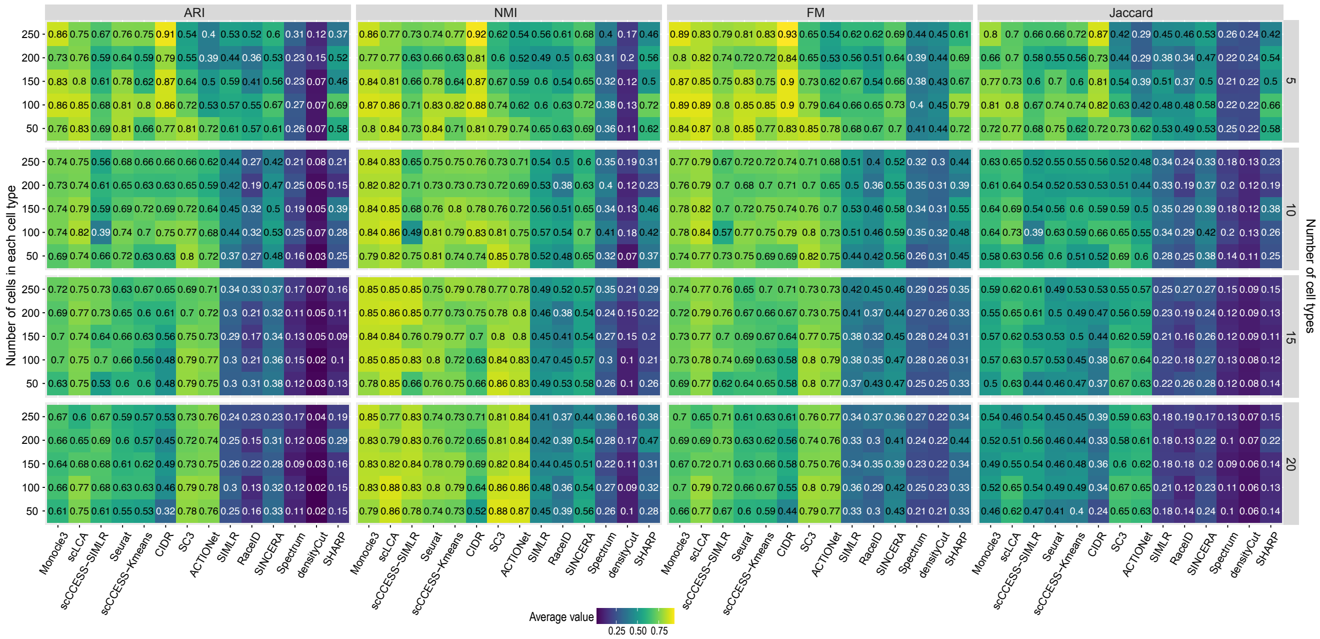

Fig S3. Concordance of clustering output and pre-defined cell type labels as quantified by four concordance measures with different true number of cell types ranges (i.e. 5, 10, 15, and 20) and different number of cells in each cell type (i.e. 50, 100, 150, 200, 250). Each tile represents the average score across 10 tests.

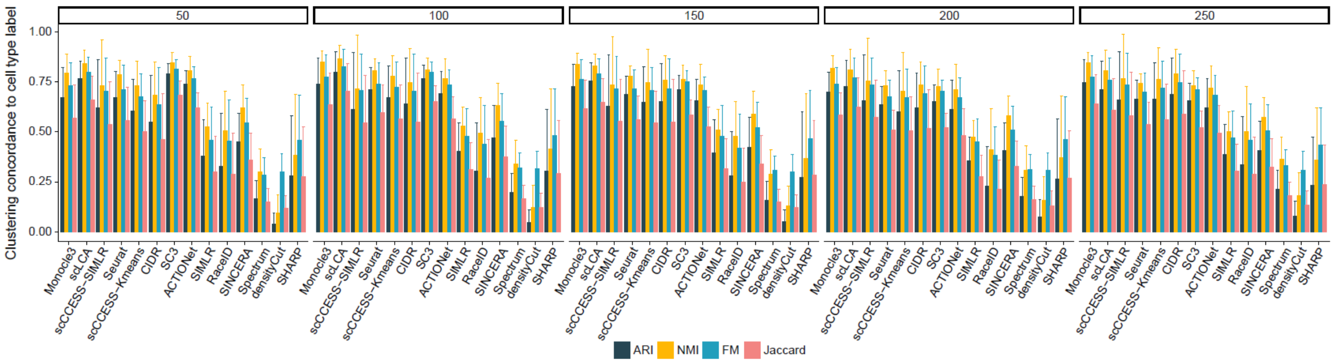

Fig S4. Concordance of clustering output and pre-defined cell type labels quantified by four concordance measures for datasets with different number of cells in each cell type (i.e. 50, 100, 150, 200, and 250). Error bars represent the standard deviation.

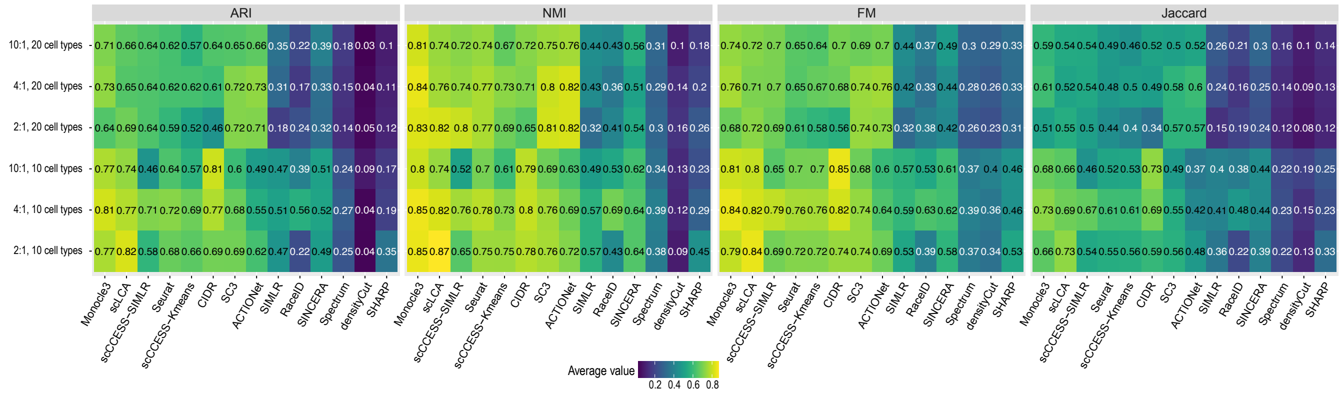

Fig S5. Comparison of cell clustering performance of 14 methods on datasets with different imbalance ratio (i.e., 2:1, 4:1, and 10:1) and different number of cell types (i.e., 10 and 20). Each tile represents the average score across 10 tests.

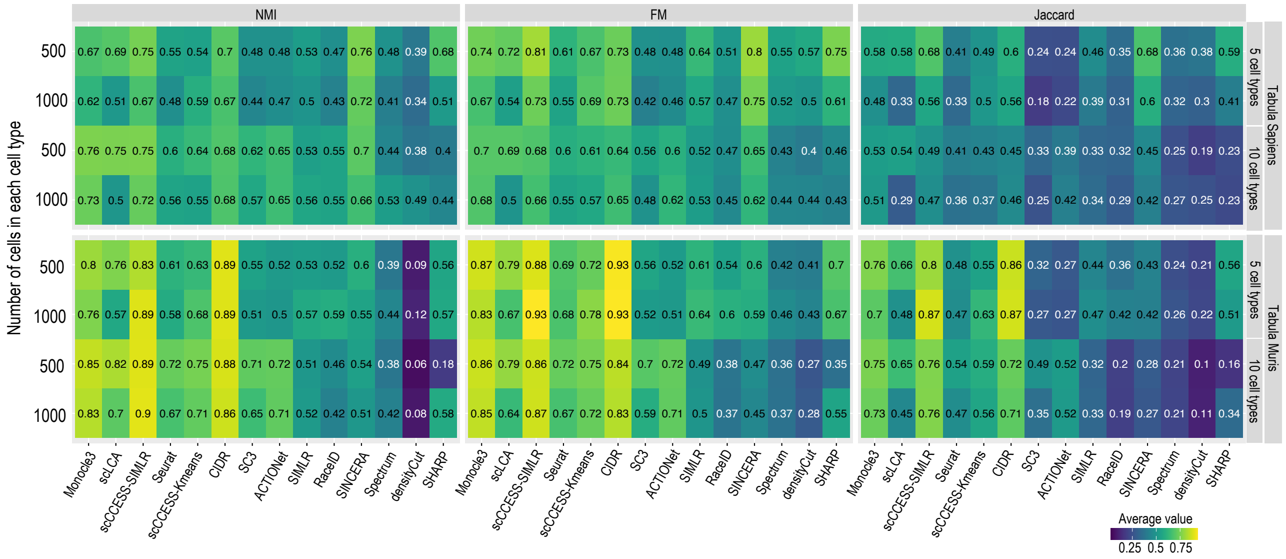

Fig S6. Comparison of cell clustering performance of 14 methods on datasets with large numbers of cells sampled from Tabula Sapiens and Tabula Muris. Each tile represents the average score across 10 tests.

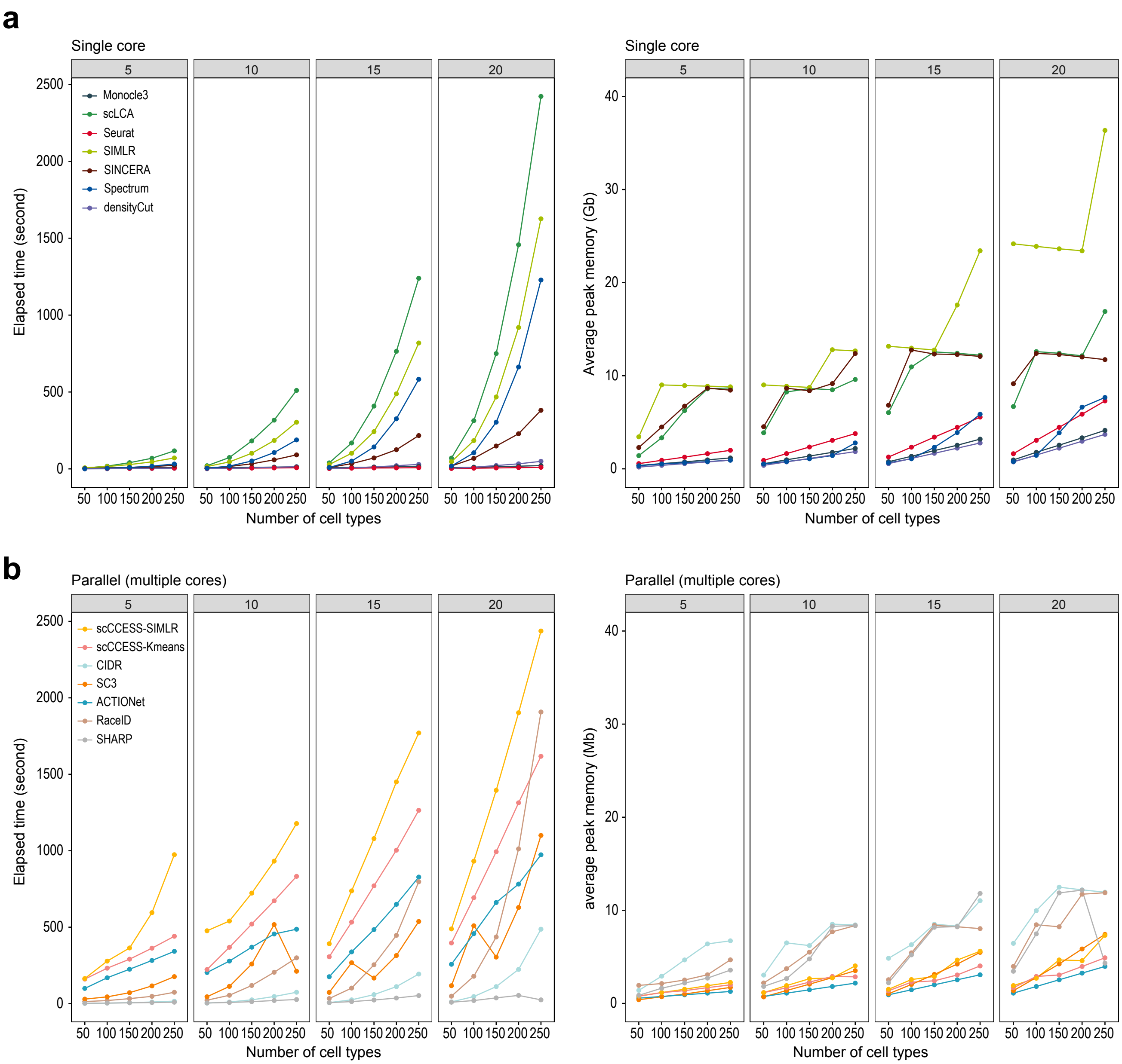

Fig S7. Benchmark of the elapsed time and peak memory usage on datasets with different number of cells (i.e., 50, 100, 150, 200, 250) and different number of cell types (i.e., 5, 10, 15, and 20).

(a) The running time and peak memory usage of methods that uses only a single CPU core (i.e., densityCut, scLCA, SIMLR, Monocle3, Seurat, Spectrum, and SINCERA).

(b) The running time and peak memory usage of methods that employ multi-cores for parallel computing (i.e., ACTIONet, RaceID, scCCESS-Kmeans, SHARP, CIDR, SC3 and scCCESS-SIMLR.

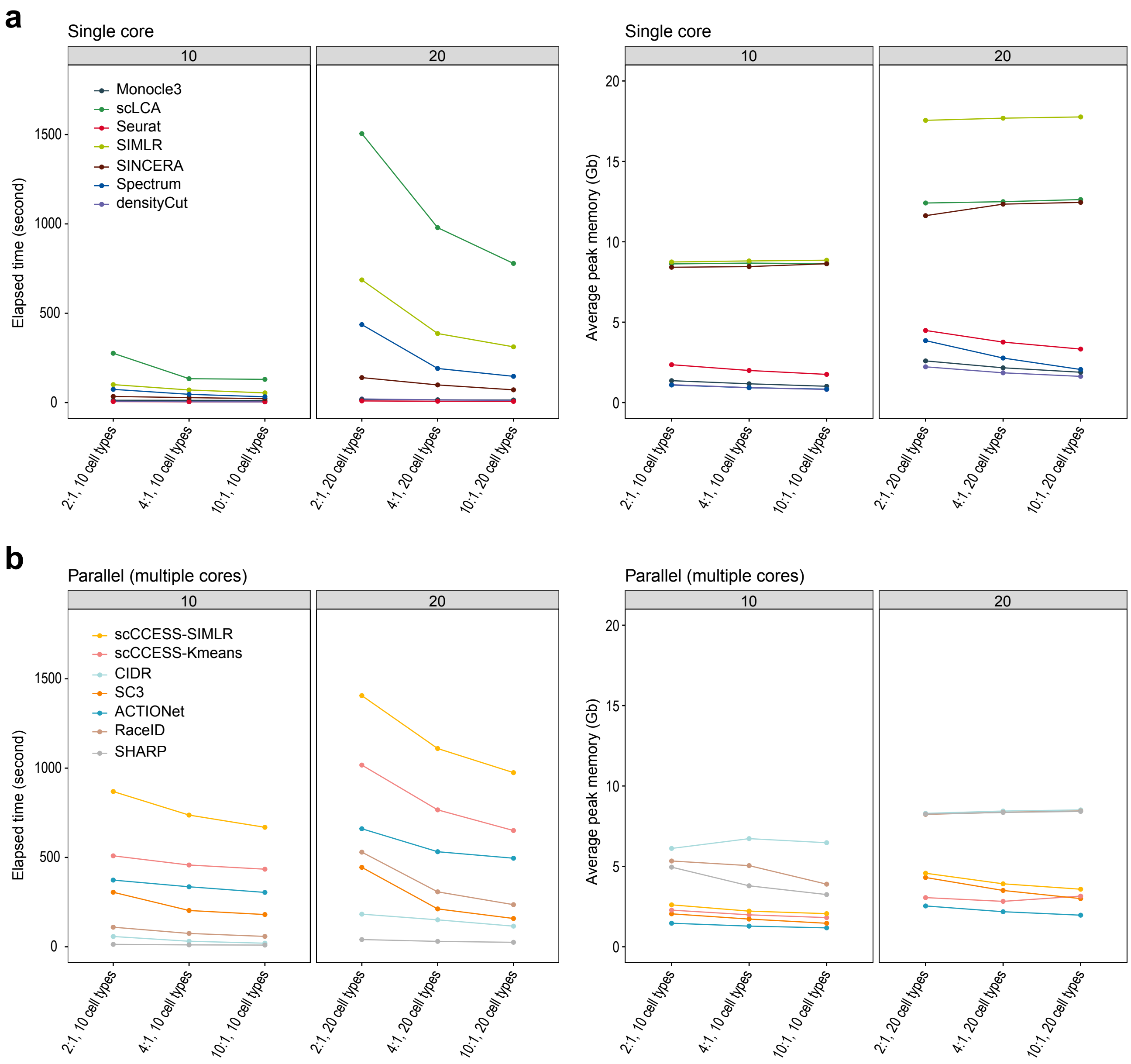

Fig S8. Benchmark of the elapsed time and peak memory usage on datasets with different imbalance ratios (i.e., 2:1, 4:1, and 10:1) and different number of cell types (i.e., 10 and 20).

(a) The running time and peak memory usage of methods that uses only a single CPU core (i.e., densityCut, scLCA, SIMLR, Monocle3, Seurat, Spectrum, and SINCERA).

(b) The running time and peak memory usage of methods that employ multi-cores for parallel computing (i.e., ACTIONet, RaceID, scCCESS-Kmeans, SHARP, CIDR, SC3 and scCCESS-SIMLR.

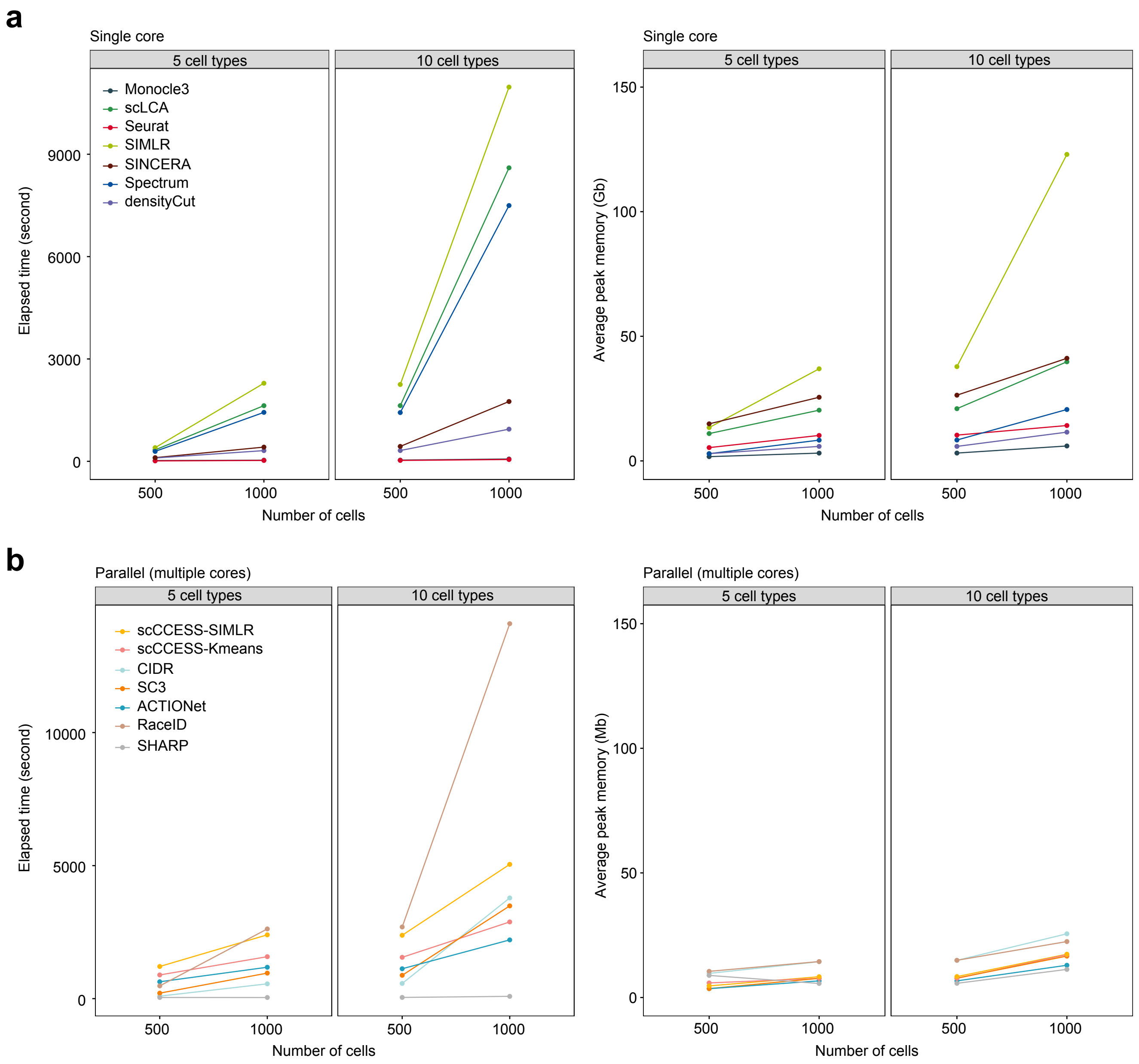

Fig S9. Benchmark of the elapsed time and peak memory usage on datasets with large number of cells.

(c) The running time and peak memory usage of methods that uses only a single CPU core (i.e., densityCut, scLCA, SIMLR, Monocle3, Seurat, Spectrum, and SINCERA).

(d) The running time and peak memory usage of methods that employ multi-cores for parallel computing (i.e., ACTIONet, RaceID, scCCESS-Kmeans, SHARP, CIDR, SC3 and scCCESS-SIMLR.
