# Supplementary material for: Benchmarking clustering algorithms on estimating the number of cell types from single-cell RNA-sequencing data
Source: Genome Biol. 2022 Feb 8;23:49. doi: 10.1186/s13059-022-02622-0 (PMC8822786; doi:10.1186/s13059-022-02622-0)
Supplement: Supplementary file 2 — Additional file 2. Review history. [file 13059_2022_2622_MOESM2_ESM.docx]

Review History

**First round of review**

**Reviewer 1**

**Are you able to assess all statistics in the manuscript, including the appropriateness of statistical tests used?**

Yes, and I have assessed the statistics in my report.

**Comments to author:**

In this manuscript, Yu et al. provides a systematic and detailed evaluation of clustering methods that estimate the number of cell types in single-cell RNA-seq data. The study used single-cell data from Tabula Muris mouse cell atlas, and various test scenarios were created by mixing a different number of cell types at different abundance. Overall, the benchmark study is of general interest to readers working on scRNA-seq since clustering is a fundamental analytical task for scRNA-seq and the number of clusters is a critical component that is often overlooked. Interestingly, the authors found that the performance of grouping and classification and the performance of identifying the correct number of cell types are not always associated, suggesting that the assessment of the clustering method with metrics on group concordance only may be misleading.

Overall, the study provides a detailed and comprehensive comparison among different methods focusing on their performance to estimate the number of cell types in the single-cell data. The paper offers a guide much needed for users and researchers who want to make a rational choice for their next single-cell clustering analysis. The paper also provides helpful information to the developer on the future directions of improvements. That said, I have the following major concerns about the study:

The authors rely on the Tabula Muris dataset for the entire evaluation, which is a major limitation in the experimental design. I suggest the authors to explore additional datasets to reach a robust conclusion.

There is no statistical test to support the statement or observation in several comparisons. For example, on page 9, lines 55-59, there is a statement: "While some methods such as scLCA, scCCESS-SIMLR and scCCESS-Kmeans appear to under-estimate the number of cell types when the imbalanced ratio increases (Fig. 5a), most other methods show no clear changes in performance." However, I feel that there is a general trend that the performance of each method tested drops when the ratio of imbalance increases.

It would be great to have a thorough discussion section, with a detailed analysis of several trends the author identified. For example, why community detection method performs better in general? Why do some methods perform poorly on the number of types while scored well in concordant metrics, especially in scenarios with equal number of cells in each cluster?

Additional minor comments:

Page 1, line 27. "The lack of benchmark studies however complicate the choice of the methods.", complicate → complicates.

Page 5, line 54. Figure 2 legend. "Each bar represent" → "Each bar represents"

Figure 4. It is a challenge for human eyes to match each line plot with the corresponding methods. Please add some visual aides to the presentation.

**Reviewer 2**

**Are you able to assess all statistics in the manuscript, including the appropriateness of statistical tests used?**

Yes, and I have assessed the statistics in my report.

**Comments to author:**

The authors systematically benchmark a range of popular clustering algorithms on estimating the number of cell types in a variety of settings. This is timely and useful since not very many comparisons have been done in this area. The paper is well thought out and well written. The language is very clear. The experiments are well designed and informative.

The methods are appropriate to the aims.

The criteria used are well chosen and informative. The results are summarized in Fig 7 which is excellent. As in most cases, there is no clear winner, and one will need to choose a method based on the problem and data available. I like that the authors do not pick one or two methods to recommend but rather present a table with strengths and weaknesses for each method.

Overall, the paper is very good and will be very useful once published.

However, I would like to recommend the following changes before publication:

- The range of cell types explored is only from 5 to 20. While is reasonable, more recently there has been a lot of interest in single cell experiments involving more cell types. Some discussion about this is necessary. Also, possibly expanding the upper limit.

- The range of cells in the bias experiments is limited to 250. Recent work has involved experiments with tens of thousands of cells. I would recommend adding some more experiments to investigate the performance of these methods with much more cell

- A literature search identified some methods that are not included here. One is well known - SINCERA. I suggest including this as well.

- Another existing method, described in PMID: 32703984, does not have a name as such, but appears to be better than several of the ones included here (Seurat, SC3, RaceID). Interestingly, this paper also includes a comparison of 7 methods on some of the criteria used here. I would suggest including this one too, as well as discussing whether the comparison done there is consistent with the comparison in this paper, for the common methods.

**Authors Response**

**Point-by-point responses to the reviewers’ comments:**

Reviewer #1:

In this manuscript, Yu et al. provides a systematic and detailed evaluation of clustering methods that estimate the number of cell types in single-cell RNA-seq data. The study used single-cell data from Tabula Muris mouse cell atlas, and various test scenarios were created by mixing a different number of cell types at different abundance. Overall, the benchmark study is of general interest to readers working on scRNA-seq since clustering is a fundamental analytical task for scRNA-seq and the number of clusters is a critical component that is often overlooked. Interestingly, the authors found that the performance of grouping and classification and the performance of identifying the correct number of cell types are not always associated, suggesting that the assessment of the clustering method with metrics on group concordance only may be misleading.

Overall, the study provides a detailed and comprehensive comparison among different methods focusing on their performance to estimate the number of cell types in the single-cell data. The paper offers a guide much needed for users and researchers who want to make a rational choice for their next single-cell clustering analysis. The paper also provides helpful information to the developer on the future directions of improvements. That said, I have the following major concerns about the study:

*Response: We thank the reviewer for the positive comments and suggestions. We have addressed all raised points below.*

1. The authors rely on the Tabula Muris dataset for the entire evaluation, which is a major limitation in the experimental design. I suggest the authors to explore additional datasets to reach a robust conclusion.

*Response: We thank the reviewer for this suggestion. The reason for relying on Tabula Muris dataset is due to its diverse and large number of cell types and number of cells from these cell types which allow us to create various experimental settings and scenarios for assessing and benchmarking different aspects/characteristics of the clustering algorithms.*

*We agree that it is important to evaluate if results obtained from Tabula Muris data are reproducible using data from other sources. We have now performed additional evaluation using data from Tabula Sapiens (a.k.a. Human Cell Atlas) project. In particular, we assessed the clustering algorithms on estimating the number of cell types, cell clustering, and computation expenses from Tabula Sapiens data with different numbers of cell types (i.e., 5 and 10) and different numbers of cells in each cell type (i.e., 500 and 1000). To enable direct comparison, we have also performed the same benchmark on Tabula Muris data. We found that, other than an exception of the SINCERA method, results of each method from using Tabula Sapiens data are largely consistent with those from using Tabula Muris. We included these results in the new Fig. 6 and Fig. S6 and S9 and revised the manuscript, including adding a new section “Cross-comparison on datasets with high cell numbers using Tabula Muris and Tabula Sapiens data”.*

*As another validation, we have also compared our results with a recent report (PMID: 32703984) that has compared some clustering algorithms in terms of estimating the number of cell types on a few datasets that are independent from the datasets we have used in this study. We found, where the comparisons overlapped, that the results are largely consistent. For example, in PMID: 32703984, SC3 appears to be significantly over-estimating the number of clusters in multiple cases (e.g., datasets “Klein” and “Patel”) and RaceID takes significantly more computation time compared to methods such as Seurat and SC3. These additional comparisons strengthen the conclusions we have drawn previously. We have incorporated these results in the discussion section (second paragraph) of the revised manuscript.*

2. There is no statistical test to support the statement or observation in several comparisons. For example, on page 9, lines 55-59, there is a statement: "While some methods such as scLCA, scCCESS-SIMLR and scCCESS-Kmeans appear to under-estimate the number of cell types when the imbalanced ratio increases (Fig. 5a), most other methods show no clear changes in performance." However, I feel that there is a general trend that the performance of each method tested drops when the ratio of imbalance increases.

*Response: We appreciate this comment. For the analysis of impact in terms of the increasing number of cell types and the increasing number of cells in each cell type, we have now performed linear regression and analysed the slopes of the fitted lines for determining their statistical significance in support of the trends/biases we identified for the clustering methods. These results are now included in the new Fig. 4 and the texts are updated accordingly (see Section “Bias analysis of clustering algorithms on estimating the number of cell types”).*

*For Fig. 5a, we have now reorganised the plot by grouping results from each method together. We found this presentation helps highlight the trends of each method with respect to the changing imbalanced ratios of major and minor cell types and have revised the manuscript for these results. See section “Impact of imbalance of cell ratios on number of cell type estimation and clustering”, second paragraph.*

3. It would be great to have a thorough discussion section, with a detailed analysis of several trends the author identified. For example, why community detection method performs better in general? Why do some methods perform poorly on the number of types while scored well in concordant metrics, especially in scenarios with equal number of cells in each cluster?

*Response: We thank the reviewer for these comments. As suggested, we have now included our opinions on these points in the discussion section (second paragraph) in the revised manuscript.*

4. Page 1, line 27. "The lack of benchmark studies however complicate the choice of the methods.", complicate → complicates.

*Response: This has now been corrected in the revised manuscript.*

5. Page 5, line 54. Figure 2 legend. "Each bar represent" → "Each bar represents"

*Response: This has now been corrected in the revised manuscript.*

6. Figure 4. It is a challenge for human eyes to match each line plot with the corresponding methods. Please add some visual aides to the presentation.

*Response: We acknowledge this and have now updated the figure so that the method legends are ordered according to the “median value of deviation” of each method at the last point on the x-axis (i.e., 20 cell types) in panel (a) (see Figure in response to the above point #2). We hope this aids the match of each line in the plot to the corresponding method.*

Reviewer #2:

The authors systematically benchmark a range of popular clustering algorithms on estimating the number of cell types in a variety of settings. This is timely and useful since not very many comparisons have been done in this area. The paper is well thought out and well written. The language is very clear. The experiments are well designed and informative. The methods are appropriate to the aims. The criteria used are well chosen and informative. The results are summarized in Fig 7 which is excellent. As in most cases, there is no clear winner, and one will need to choose a method based on the problem and data available. I like that the authors do not pick one or two methods to recommend but rather present a table with strengths and weaknesses for each method.

*Response: We thank this reviewer for the positive comments on our work. We have addressed all suggestions point by point below.*

1. The range of cell types explored is only from 5 to 20. While is reasonable, more recently there has been a lot of interest in single cell experiments involving more cell types. Some discussion about this is necessary. Also, possibly expanding the upper limit.

*Response: We thank the reviewer for this comment and agree that datasets with increasingly large numbers of cell types are being generated recently with improving capability of scRNA-seq techniques, potentially with more complex structure such as cell type hierarchy. As suggested, we have now acknowledged this as a potential limitation and included the following in the discussion section (third paragraph):*

*“Although the sampling framework we used in this study creates scRNA-seq data with discrete cell types from the Tabula Muris resource, cell type structures from many biological systems are hierarchical with subpopulations of cells residing in each major cell type [37]. Related to this, the improving capability of scRNA-seq for profiling complex tissues and organs has led to datasets with increasing numbers of cell types and potentially further compounded by hierarchical cell type relationships. Thus, developing frameworks that are capable of benchmarking multi-resolution or multi-scale clustering on datasets with large numbers of cell types is a critical future research direction.”*

2.. The range of cells in the bias experiments is limited to 250. Recent work has involved experiments with tens of thousands of cells. I would recommend adding some more experiments to investigate the performance of these methods with much more cell

*Response: As suggested, we have now included in our benchmark datasets with 2500 to 10000 cells. In particular, we sampled either 500 or 1000 cells from either 5 or 10 cell types in Tabula Muris and Tabula Sapiens data. This allows us to cross-compare the performance of clustering algorithms on datasets with high cell numbers from two different data sources. These results are now included in the new Fig. 6 and Fig. S6 and S9 and the manuscript have been revised to incorporate these new results (including a new section on “Cross-comparison on datasets with high cell numbers using Tabula Muris and Tabula Sapiens data”).*

3.. A literature search identified some methods that are not included here. One is well known - SINCERA. I suggest including this as well.

*Response: We thank this suggestion. We have now included SINCERA in our evaluation framework. In particular, we have updated all figures (main figures 1-8 and supplementary figures 1-9) and revised the manuscript accordingly to incorporate these new results.*

4. Another existing method, described in PMID: 32703984, does not have a name as such, but appears to be better than several of the ones included here (Seurat, SC3, RaceID). Interestingly, this paper also includes a comparison of 7 methods on some of the criteria used here. I would suggest including this one too, as well as discussing whether the comparison done there is consistent with the comparison in this paper, for the common methods.

*Response: We appreciate this suggestion. We have referred to the publication by Peyvandipour et al., (Scientific Reports; PMID: 32703984), but the authors did not provide any implementation (code or package) for their method. Given their study also compared a few methods for estimating the number of cell types and computation times, we have now compared and discussed these results in our revised manuscript (Discussion section, second paragraph).*

**Second round of review**

**Reviewer 1**

The authors have substantially revised the manuscript and satisfactorily addressed my concerns in the initial review. I have no additional questions for the authors.
